# Annotations

**Published:** 1910-05-14

**Authors:** 


### Annotations.

### Goethe's Last Illness.

In the current number of the *Polyclinic* appears a short article by Sir Jonathan Hutchinson on Goethe's last illness, which in respect of its suddenness was very similar to that of King Edward. Goethe became ill while apparently in the best of health, and died at the advanced age of eighty-three years after less than a week's illness. His fatal indisposition began with a chill which developed, as Sir Jonathan suggests, into catarrhal pneumonia with pleural effusion. This diagnosis is largely a guess, though the recorded symptomatology agrees with it. As an alternative one might suggest that he died of cerebral trouble, probably a small clot, as in the case of the late Queen Victoria. The rigor and pain some days before his death are much less easy to explain, and the theory of pleural effusion seems to agree best with the signs. The old philosopher, whose last words "More light" have often been quoted, was aphasic some time before his death, but apparently quite conscious, and is said to have fallen asleep and to have passed away in slumber. No autopsy was made, and the diagnosis, as in the case of Mozart and Schumann's last illness, must be a matter of speculation, but the clinical phenomena that have been placed on record are more full and complete than in the case of the two musicians, so that Sir Jonathan Hutchinson's conclusions are probably correct.

### The Antiquity of Spermaceti.

Dr. Emil Reich's recent interesting letter in the *Times* concerning the literature of spermaceti was scarcely necessary to prove to anyone with the slightest knowledge of medical history the absurdity of the statement alleged to have been made by the opponents of Herr Bode in the now notorious Da Vinci bust controversy. We say alleged advisedly, for we can scarcely believe that a German analytical chemist, who is, after all, a member of a highly trained and encyclopædically intelligent class, would have been so crassly ignorant of pharmaceutical and chemical history as to have committed himself to the declaration that "spermaceti was unknown before 1700." We owe the interesting bits of information that have been published since this astounding dictum was made known probably to the misdirected enthusiasm of an artist-journalist whose ignorance about such matters as wax and natural history is, after all, pardonable. Readers of old-time Pharmacopœias will readily agree with Dr. Reich that a cursory inquiry should reveal many references to spermaceti prior to 1700. This substance was certainly known to Paracelsus, and it is a nice point for discussion whether among the basis for unguents mentioned and used by Paulus, Avenzoar, and even by our fine old, forgotten Bullein (who should be rescued from his undeserved oblivion as soon as possible), spermaceti is not included. A little more research and a little less assertion in such matters will be desirable on the part of the Lucasians if they wish to overthrow the claims of Leonardo. At any rate Dr. Bode's critical reputation is not to be shaken by trying to prove that if the Italian painter used spermaceti he was guilty of an unpardonable anachronism.

### Industrial Employment of Mothers.

The extraordinarily complicated nature of the problems which beset the sociologist and the hygienist in connection with the improvement of the national health, more especially as it affects the nation's infants, is well shown by a report on the Industrial Employment of Married Women and Infantile Mortality, presented to the Health Department of the City of Birmingham as the outcome of a special research in some of the poorest quarters of that town. Take, for instance, the vexed question of whether mothers of families should go out to work in factories and similar places. The report shows statistically that in Birmingham the type of industrial employment in vogue does not appreciably influence the health of the mother or her infant when the standard of comparison is that of women in equally poor circumstances who are not employed industrially. In so far as it militates against breast-feeding, such employment has a distinctly harmful influence, clearly shown in the mortality and weight tables; but against this must be set a countervailing advantage in the better nutrition of both mother and infant resulting from the extra average wages secured by the household. It appears to be a question, says the Report, whether the additional poverty which would be occasioned by State interference to prevent mothers working for six months after a birth would not be the greater of two evils. Probably the factory or workshop employment is itself not more exacting to a pregnant woman than the daily home duties of the mother of a family. Then, again, it is pointed out that in not a few cases the factor determining employment or non-employment is the strength of character and general health of the woman herself.

### Chemists' Exhibition.

The Chemists' Exhibition, which is the sixteenth of the series organised annually by the *British and Colonial Druggist*, was opened at the Royal Horticultural Hall on May 9 and remains open until May 13. Over 80 firms of manufacturing chemists, wholesale druggists, dealers in druggists' sundries, surgical appliances, etc., took advantage of this opportunity to display their products to the numerous pharmaceutical visitors from London and the provinces. The latest improvements in pharmaceutical methods were represented at various stands, and, as might be expected, preparations of *Bacillus bulgaris* and other lacto-bacilli were among the exhibits. There were also shown a variety of bacterial vaccines and therapeutic serums. A good proportion of the exhibits were naturally devoted to such things as mineral waters, sponges, perfumery, toilet preparations, and the hundred and one articles which form part of the modern pharmacist's stock-in-trade.

